# Increasing Necking Strain through Corrugation: Identifying Composite Systems That Can Benefit from Corrugated Geometry

**DOI:** 10.3390/ma13225175

**Published:** 2020-11-17

**Authors:** Mark Fraser, Hatem Zurob, Peidong Wu, Olivier Bouaziz

**Affiliations:** 1 Department of Materials Science and Engineering, McMaster University, 1280 Main St. W., Hamilton, ON L8S 4L7, Canada; frasem3@mcmaster.ca; 2 Department of Mechanical Engineering, McMaster University, 1280 Main St. W., Hamilton, ON L8S 4L7, Canada; peidong@mcmaster.ca; 3 Laboratoire d’Etude des Microstructures et de Mécanique des Matériaux (LEM3), CNRS, Université de Lorraine, Arts et Métier Paris Tech, F 57000 Metz, France; olivier.bouaziz@univ-lorraine.fr; 4 LABoratoire d’EXcellence DAMAS, Université de Lorraine, F 57000 Metz, France

**Keywords:** composite materials, architectured materials, corrugated reinforcements, finite element modeling (FEM), mechanical properties

## Abstract

Under some circumstances, composites with a corrugated reinforcement geometry show larger necking strains compared to traditional straight reinforced composites. In this work, finite element modeling studies were performed for linearly hardening materials, examining the effect of material parameters on the stress–strain response of both corrugation and straight-reinforced composites. These studies showed that improvements in necking strain depend on the ability of the corrugation to unbend and to provide a boost in work hardening at the right time. It was found that there is a range of matrix yield strengths and hardening rates for which a corrugated geometry will improve the necking strain and also a lower threshold of reinforcement yield strength below which no improvement in necking strain is possible. In addition, benefit maps and surfaces were generated that show which regions of property space benefit through corrugation and the corresponding improvement in necking strain that can be achieved.

## 1. Introduction

Many forming processes are limited by the maximum uniform deformation or the necking strain. The necking strain of a material can be increased by introducing a wavy or corrugated reinforcement. There have been a number of studies that investigate the effect of a corrugated geometry on the behavior of isolated corrugations [1,2,3,4,5,6], sandwich panels with a corrugated core [7,8,9], and composites materials with corrugated reinforcements [3,4,10,11,12,13,14,15,16,17,18,19,20,21,22]. Dayyani et al. [1] and Thill et al. [2] both explored the behavior of isolated trapezoidal corrugated structures, whereas Bouaziz [3], Boke [4], Ge [5], and Fraser et al. [6] studied and modeled the behavior of isolated sinusoidal corrugated structures. Luo et al. [7] and Gilchrist et al. [8] studied the bending of corrugated board sandwich panels, and Yokozeki et al. [9] examined sandwich panel structures with corrugated cores for the application of morphing wings in aviation. In general, these studies have shown that when loaded in tension along the longitudinal direction, the corrugated structure shows a lower initial stiffness and lower strength level, followed by a hardening process as a result of the unbending of the corrugation. In some of the studies [3,4,6,13,21,23] where the deformation was taken to high levels of strain, this unbending process was shown to lead to a material that necks at a higher level of strain compared to an uncorrugated counterpart and yet still has a comparable ultimate tensile strength. This ability to improve necking strain, without sacrificing strength, can lead to promising combinations of properties, particularly for energy absorption applications. 

With regard to the composite materials with a corrugated reinforcement geometry, it is of interest to determine under what conditions the use of a corrugated geometry improves the necking strain of the composite. The effect of material properties has been explored for corrugated composites in elastic systems by Chiskis and Parnes [12], Chou and Takahashi [13], and Khatam and Pindera [16]. Abdelrahman and Nayfeh [14] used analytical micromechanical modeling to predict the stress distribution within a corrugated fiber reinforcement in an elastic matrix, providing valuable information on the local stress state in the composite material, although the model was limited to elastic materials and did not provide results on the global stress–strain behavior of the composite. Finally, Shi et al. [21] investigated the effect of the rate sensitivity of elastic–viscoplastic corrugated reinforcements on the necking strain of the composites. Therefore, there is a gap in the knowledge regarding the effect of the material properties of both the matrix and reinforcement phases for corrugation-reinforced composites with plastically deforming materials. 

Fraser et al. [24] utilized FEM simulations to compare the unbending behavior of an isolated corrugation to that of a corrugation embedded in a matrix material, exploring how the unbending behavior was impacted by the matrix material and the geometry of the corrugation. Another investigation [25] illustrated how corrugation-reinforced composites can be used to fill a hole in the material property space characterized by high strength and high necking strain. The purpose of this paper is to further explore the effect of the plastic material properties of the matrix and reinforcement components on the necking strain of these materials and ultimately establish guidelines for determining composite systems that will benefit from corrugated reinforcement architecture. This was accomplished by performing parametric studies on the linear hardening properties of the component materials using finite element modeling (FEM) simulations.

## 2. Modeling Procedure

**Figure 1:**
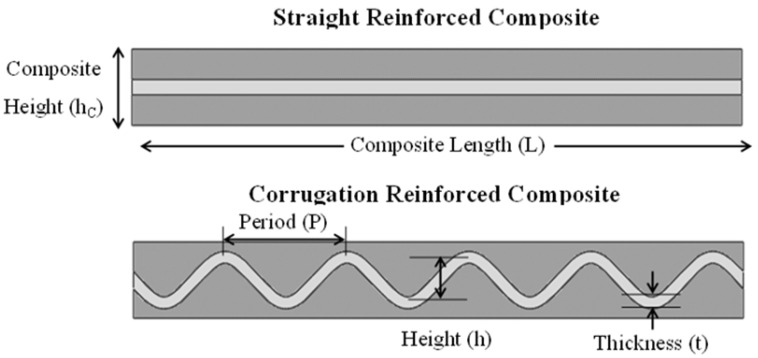
Depiction of straight reinforced composite (top) and corrugation reinforced composite (bottom) used in finite element modeling (FEM) simulations.

For each combination of matrix and reinforcement properties, two simulations were performed; the first had a single straight reinforcement, and the second had a single corrugated reinforcement, as shown in Figure 1. Regardless of the geometry, each sample had a composite length, L, of 40 mm, composite height, hc, of 5 mm and reinforcing volume fraction of 20%. The corrugation geometry itself can be described as a wave with a corrugation height, h, period, P, and thickness, t, as labeled in Figure 1. The base case that was considered for the different material systems had a corrugation height of 3 mm, period of 8 mm, and thickness of 0.78 mm. This is contrasted with the straight reinforced composite in which the reinforcement had a thickness of 1 mm. The FEM simulations were performed using two-dimensional plane strain models in ABAQUS.

The boundary conditions were defined so as to simulate tensile loading in the longitudinal direction and consisted of horizontally fixing the left end of the sample and displacing the right end of the sample by a finite amount in the horizontal direction. Additionally, the midpoints of both ends were fixed vertically to prevent the simulated part from drifting in the vertical direction. As the simulations were two-dimensional, they used first-order plane strain quadrilateral elements, CPE4. Following mesh refinement studies, the element size was selected such that the number of elements within the simulation was between 20,000 and 25,000.

Both the matrix and reinforcement materials were defined such that they showed elastic–linearly plastic behavior and thus follow the expression, σ = YS + Kε, where YS is the yield strength and K is the linear hardening rate. Linear hardening behavior was selected, as this allowed for simpler isolation of the effects yield strength and work hardening compared to the commonly employed power-law hardening. The mechanical properties of the matrix were set similar to copper with an elastic modulus of 115 GPa, a Poisson’s ratio of 0.33, yield stress in the range of 50 to 400 MPa, and hardening rates in the range of 200–1000 MPa [26,27]. The reinforcement had mechanical properties similar to those of a ferrite/pearlite steel with an elastic modulus of 207 GPa, Poisson’s ratio of 0.3, yield stress in the range of 300–1200 MPa, and hardening rates in the range of 1000–4000 MPa [26,28]. The interface between the matrix and reinforcement phases was idealized and was modeled assuming a perfect interface that would not separate. Although not realistic, this simplification was employed to limit the number of confounding variables and to isolate the effect of the material properties of the matrix and reinforcement. Future studies should be completed exploring the effect of the interfacial strength on the behavior of these composites.

Four parametric studies were performed to examine the effect of the yield strength and linear hardening rate for both components. Each study focused on a single parameter, varying that parameter across a range of values while fixing all the other material parameters. The values of these parameters for each of the studies are summarized in Table 1. The range of values selected for each parameter in each parametric study were established by first selecting an arbitrary initial parameter value, running the set of simulations with that value and then evaluating the results of those simulations. Then, subsequent sets of simulations were completed at increasingly higher values of the parameter, with each set of simulations evaluated after completion. Once it was established that the trend in behavior had leveled out and subsequent increases in the parameter would yield similar results, the highest parameter value was set as an upper bound. A similar process was completed by running subsequent sets of simulations at parameter values below the initial value until a lower bound was determined.

## 3. Results

**Figure 2:**
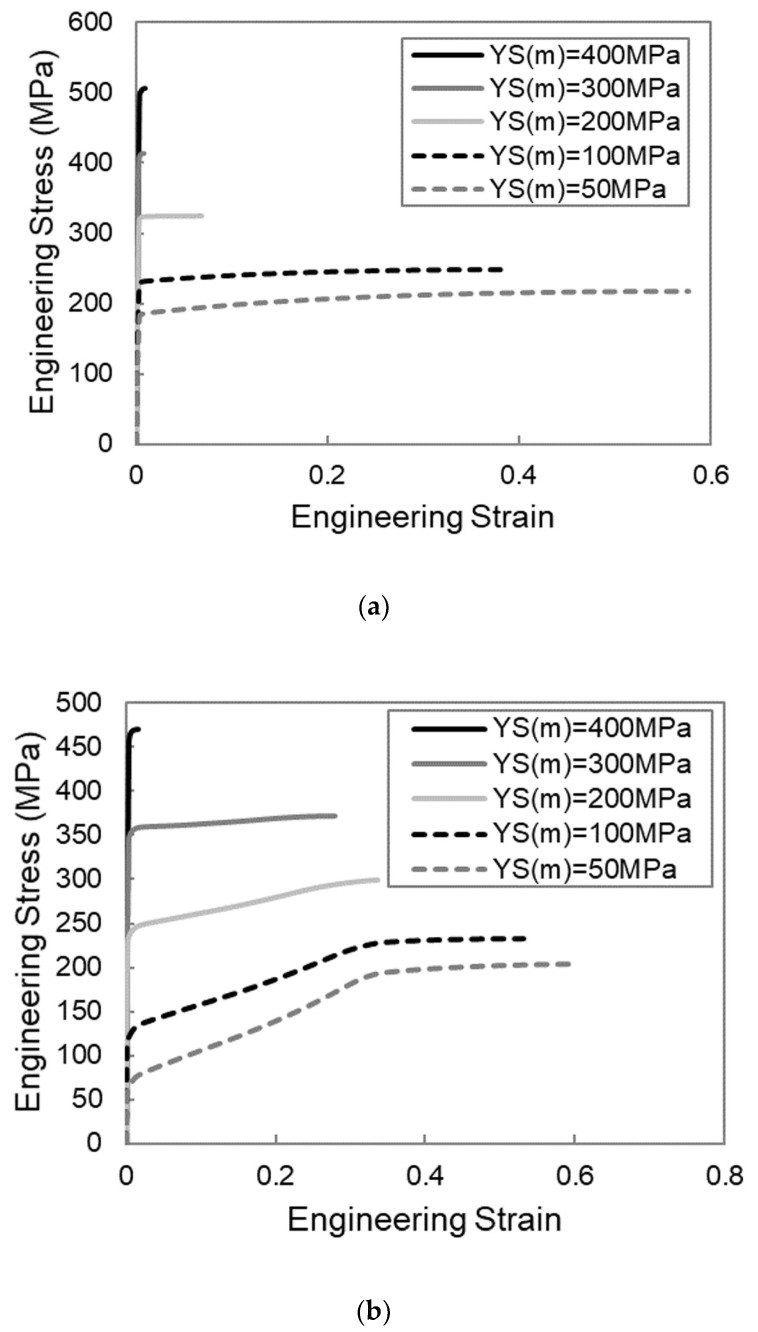

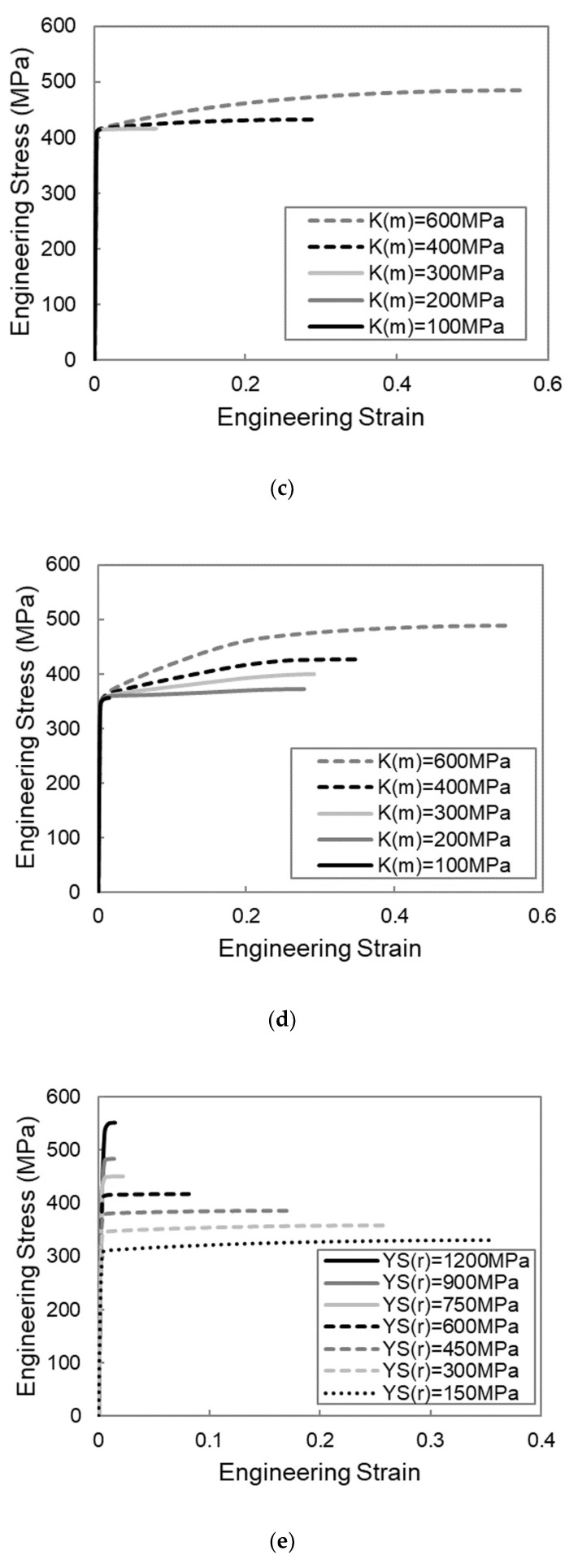

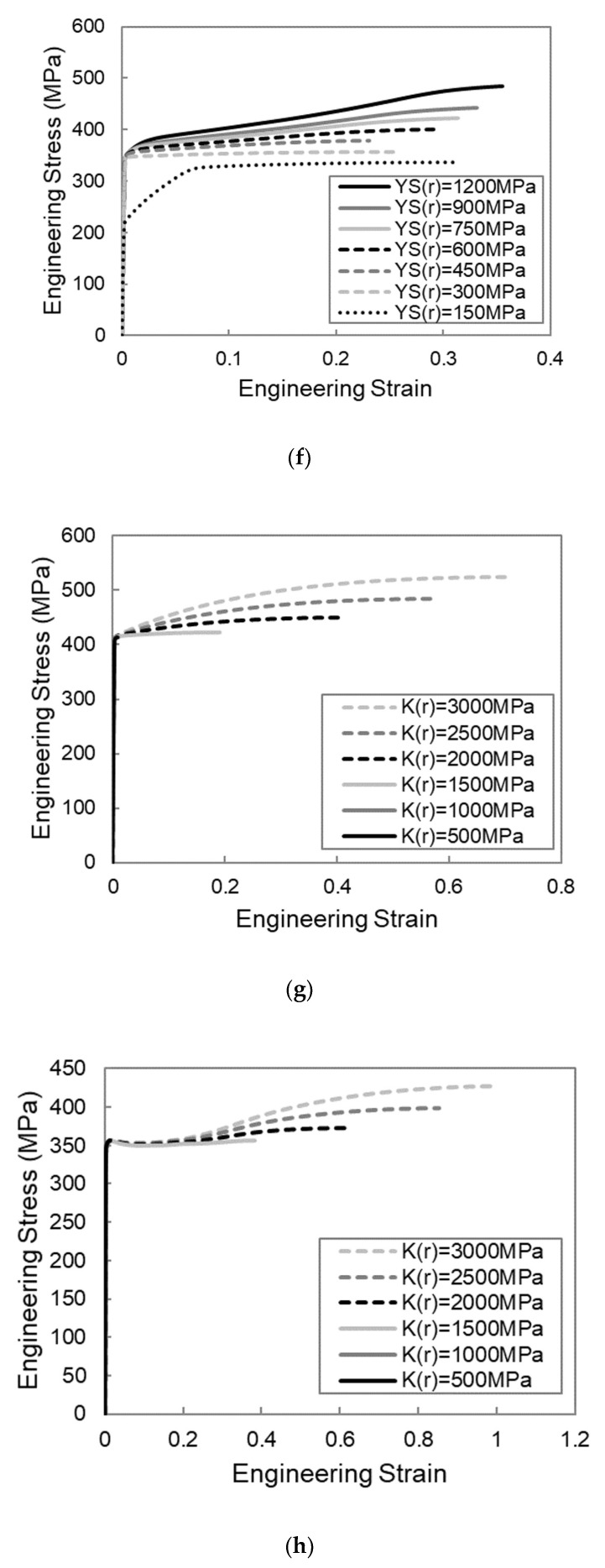
Effect of material properties on the stress–strain curves obtained using FEM simulations of straight and corrugation reinforced composites. The following material properties were varied: (**a**) matrix yield strength, YS(m), for a composite with a straight reinforcement; (**b**) matrix yield strength, YS(m), for a composite with a corrugated reinforcement; (**c**) matrix hardening rate, K(m), for a composite with a straight reinforcement; (**d**) matrix hardening rate, K(m), for a composite with a corrugated reinforcement; (**e**) reinforcement yield strength, YS(r), for a composite with a straight reinforcement; (**f**) reinforcement yield strength, YS(r), for a composite with corrugated reinforcement; (**g**) reinforcement hardening rate, K(r), for a composite with straight reinforcement and (**h**) reinforcement hardening rate, K(r), for a composite with corrugated reinforcement.

**Figure 3:**
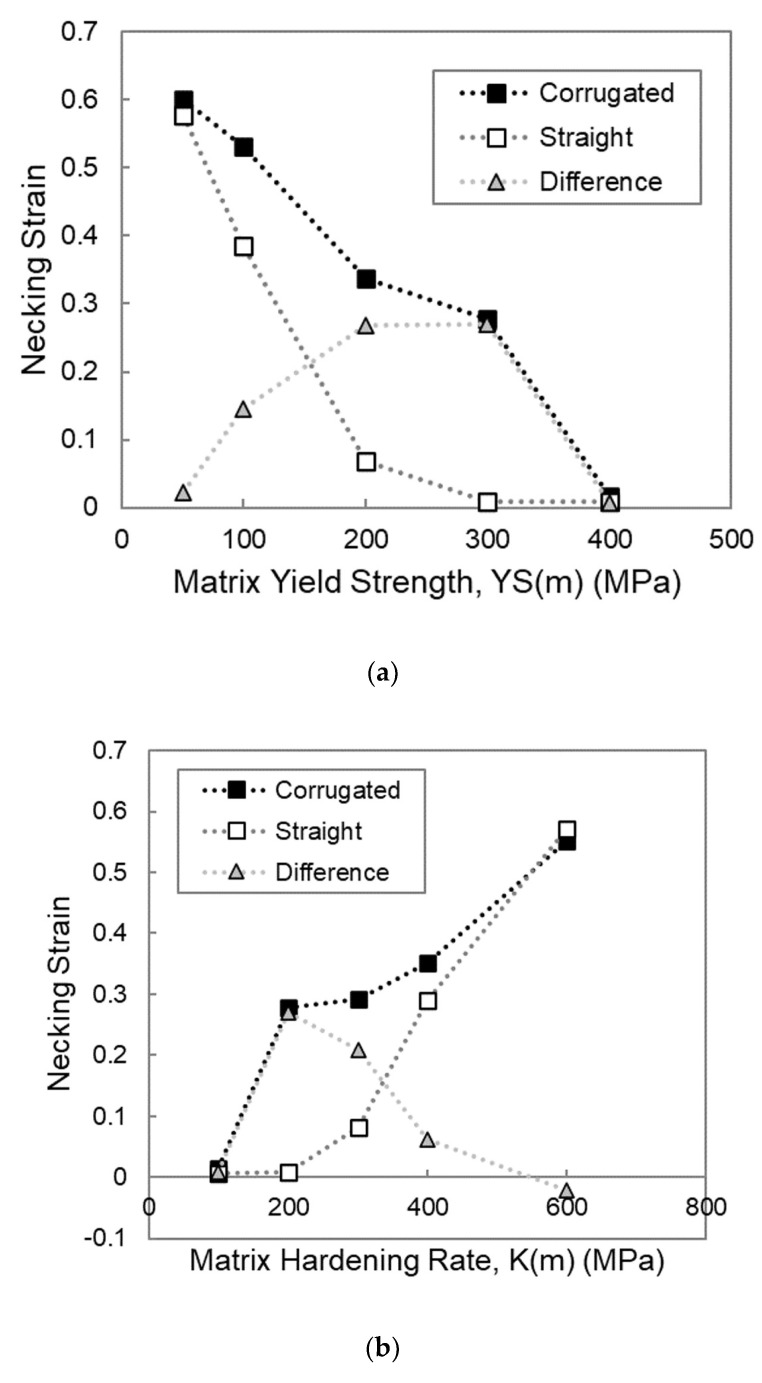

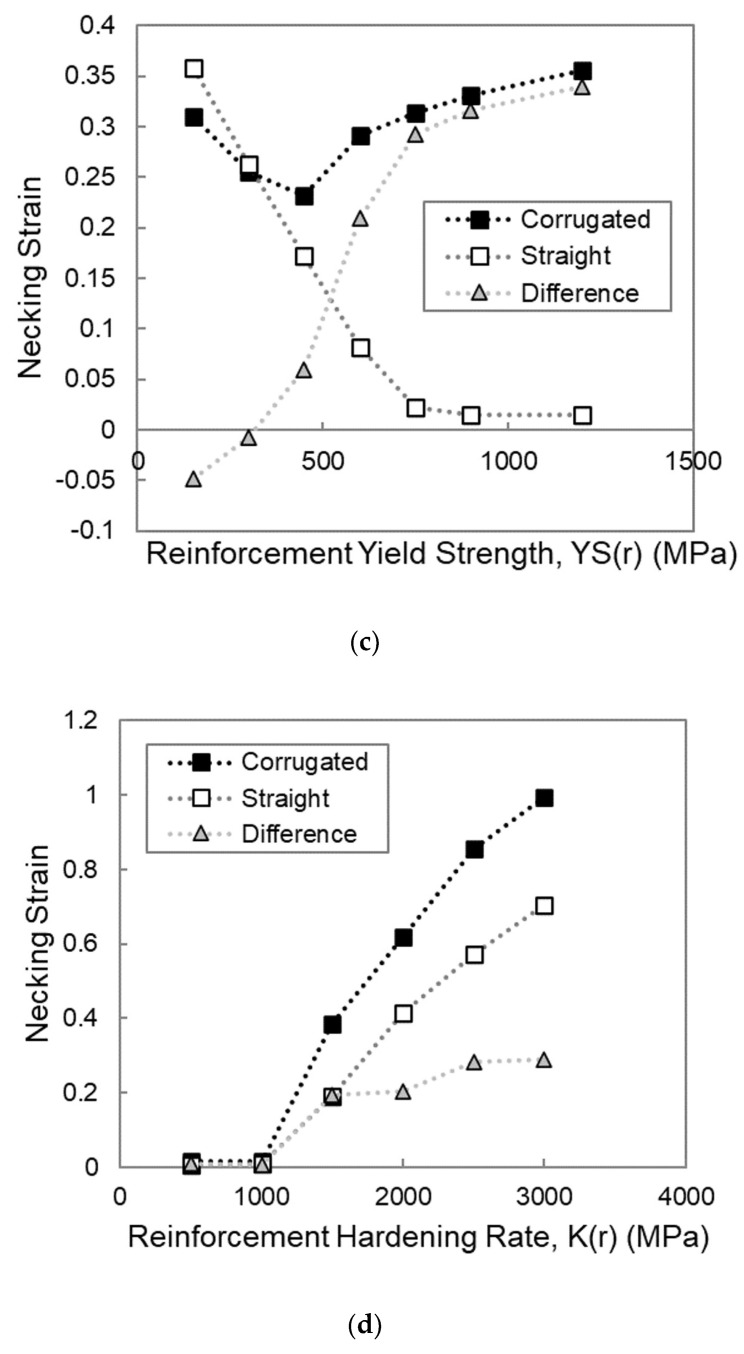
Plots of necking strain as a function of different material parameters from FEM simulations of straight and corrugation reinforced composites. The plots also include the difference in necking strain between the corrugated and straight simulations for each value of the parameter tested. The parameters include (**a**) matrix yield strength, YS(m); (**b**) matrix hardening rate, K(m); (**c**) reinforcement yield strength, YS(r); (**d**) reinforcement hardening rate, K(r).

The stress–strain response from each of the simulations outlined above is shown in the stress–strain curves in Figure 2. Given the interest in using the corrugated architecture to improve the necking strain of the composite relative to the straight case, it is useful to plot the necking strain as a function of each of the material parameters of interest for both the straight and corrugated composites, as shown in Figure 3. Each of these plots also includes a set of points that represent the difference between the necking strains of the straight and corrugated samples. This can be thought of as a plot of the benefit of corrugation.

## 4. Discussion

One of the first things to note in comparing the stress–strain curves of the corrugated composites to those of the straight composites is that it is only under some conditions that the corrugated composite is capable of outperforming the straight composites with regard to necking strain. Therefore, certain conditions must be present in order for the corrugated composite to have a greater necking strain than a straight composite made of the same materials. Looking at the plot of necking strain versus matrix yield strength in Figure 3a, it is clear that if the matrix yield strength is too low, this necking strain is very comparable to what the straight composite is able to achieve, and thus, there is minimal benefit to be found by using a corrugated geometry. Additionally, it can be seen that if the matrix yield strength exceeds a certain value, there is also no benefit through corrugation. In this case, both the corrugated and straight composites neck at a low value of strain. Therefore, there exists a range of matrix yield strengths for which there is the opportunity to increase the necking strain of the composite through the use of a corrugated reinforcement. 

**Figure 4:**
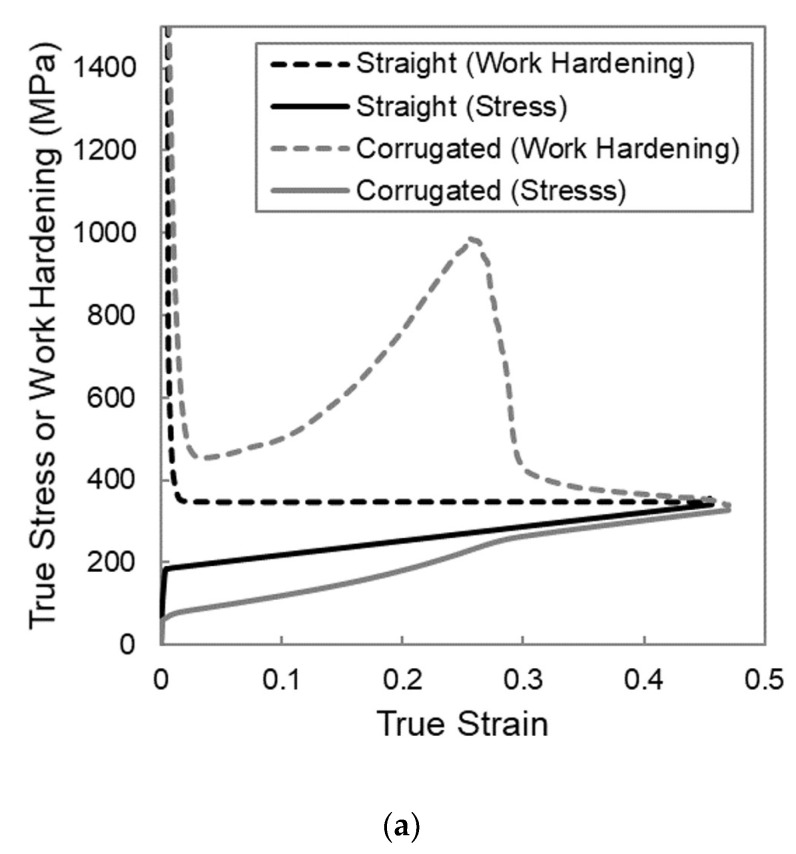

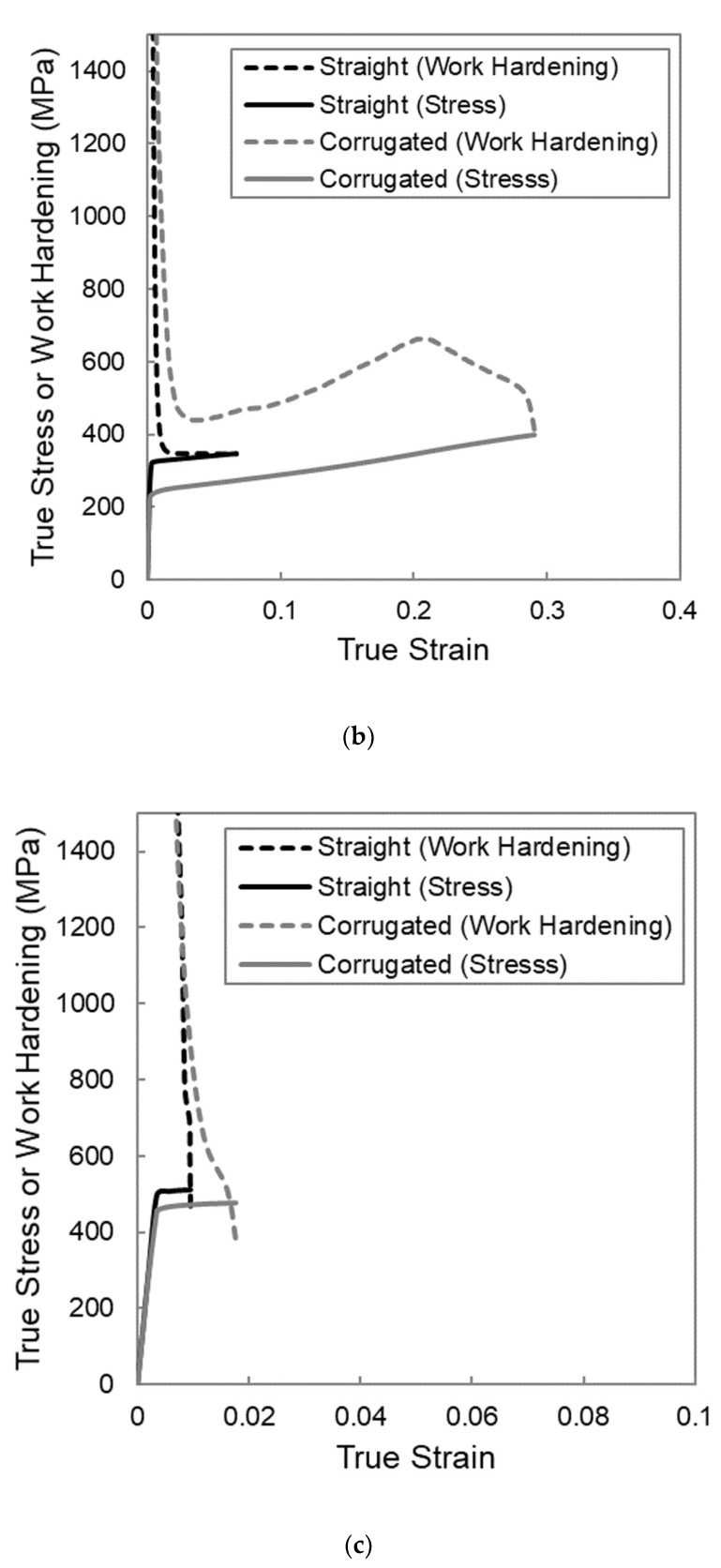
Considere-type plots of work hardening and stress as a function of strain for FEM simulations of straight and corrugation-reinforced composites made from linear hardening materials with different matrix yield strengths, YS(m); (**a**) YS(m) = 50 MPa, (**b**) YS(m) = 200 MPa, (**c**) YS(m) = 400 MPa.

To understand why these upper and lower limits exist, it is beneficial to examine the work hardening behaviour of the composite for three characteristic cases; the first case is one in which the matrix yield strength is too low, the second is one in which the corrugated composite shows a higher necking strain than the straight composite, and the third is one in which the matrix yield strength is too high. The work hardening rate of the composite was calculated by differentiating the relevant stress in Figure 2 with respect to the strain. The work hardening behavior is shown in the form of Considere-type plots in Figure 4a for YS(m) = 50 MPa, Figure 4b for YS(m) = 200 MPa, and Figure 4c for YS(m) = 400 MPa. In these plots, both the work hardening and the stress are plotted as a function of strain for each simulation. The strain at which the work hardening curve and the stress–strain curve intersect for a given simulation corresponds to the necking strain [29,30]. In the cases when the presence of the corrugated geometry improves the necking strain, there is a boost in work hardening that delays the intersection of the work hardening and stress curves and thus delays the point of necking, as shown in Figure 4b.

**Figure 5:**
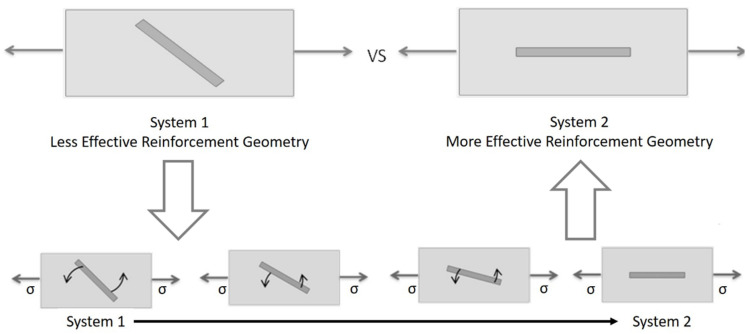
A geometric simplification of the region of the reinforcement between peak and trough and its evolution from less effective to more effective load carrying geometry representing the unbending process of a corrugation.

This boost in work hardening is a result of the evolution of reinforcement geometry that comes from the unbending of the corrugation. The corrugated reinforcement is initially at an angle to the loading direction and, therefore, it is less effective at carrying load than if it were aligned in the loading direction. Initially, deformation will be dominated by bending in the peaks and troughs, and the global stress is small. As deformation progresses, the alignment of the reinforcement transitions toward a straight alignment that is more effective at the carrying load. This evolution of the reinforcement geometry, as depicted schematically in Figure 5, strengthens the material, and it provides a boost in work hardening that can delay necking. When the matrix yield strength is too low, the boost in work hardening still occurs; however, the work hardening level returns to that of the straight reinforced composite at a strain that is smaller than the necking strain of a straight composite, and the end result is a very similar necking strain. The boost in work hardening has essentially come in too early to be of use. This is due to the fact that the low matrix yield strength results in low flow stress in the matrix relative to the hardening rate, which will cause the straight reinforced composite to have a large necking strain. To improve on the necking strain of the straight composite, the boost in work hardening would need to be larger and/or initiate at a larger strain. In the first example shown above, Figure 4a, the geometry of the corrugation was such that the boost in work hardening came in too early. On the other hand, when the matrix yield strength is too large, as in the case in Figure 4c, no boost in work hardening is apparent for the corrugated composite. This means that the corrugation was unable to unbend to any meaningful extent prior to the necking of the composite. When the matrix yield strength is high relative to its hardening rate, the ductility of the matrix is very low and is incapable of accommodating the unbending of the corrugation. In this case, both the straight and corrugated composites neck very early at a low value of strain.

Looking at Figure 3b, it appears that decreasing the matrix hardening rate has a similar effect to increasing the matrix yield strength. If the matrix hardening rate is too low, both straight and corrugated composites have low necking strains. The work hardening curves would be similar to those seen in Figure 4c when the matrix yield strength was too high, with a situation where the matrix necks prior to the unbending of the corrugation. If the matrix hardening rate is too high, both the straight and corrugated composites have large necking strains and the benefit through corrugation is minimal. This scenario shows similar work hardening curves to those seen in Figure 4a, when the matrix yield strength is too low and the boost in work hardening that comes from the unbending corrugation comes in too early. Once again, there is a range of matrix hardening rates for which a corrugated reinforcement is beneficial compared to a straight reinforcement. The interrelatedness between the effects of matrix yield strength and hardening rate can be explained by the fact that it is the flow stress of the material relative to its hardening rate that dictates when necking occurs. For linearly hardening materials, this flow stress is highly dependent on its yield strength. Using a Considere-type derivation of necking strain, ε_n_, for a linearly hardening material, it is clear that the ratio of YS(m)/K(m) is important.

(1) $$\varepsilon_{n}=1-\frac{YS}{K}$$

Shifting the focus to the reinforcement yield strength, highlighted in Figure 3c, the effect seems to be different than that observed for the matrix parameters. Instead of having a range of values for which using a corrugated reinforcement is beneficial, there appears to be a threshold value that needs to be exceeded in order for the necking strain of the corrugated composite to be larger than that of the straight composite. When the reinforcement yield strength is increased above this threshold value, the necking strain of the straight composite decreases, whereas the necking strain of the corrugated composites increases, thus increasing the benefit through corrugation. At very high levels of reinforcement yield strength, this improvement seems to level off. On the other hand, when the reinforcement yield strength is below this threshold value, both the straight and corrugated composites show large values of necking strain, with no attainable benefit through corrugation. 

**Figure 6:**
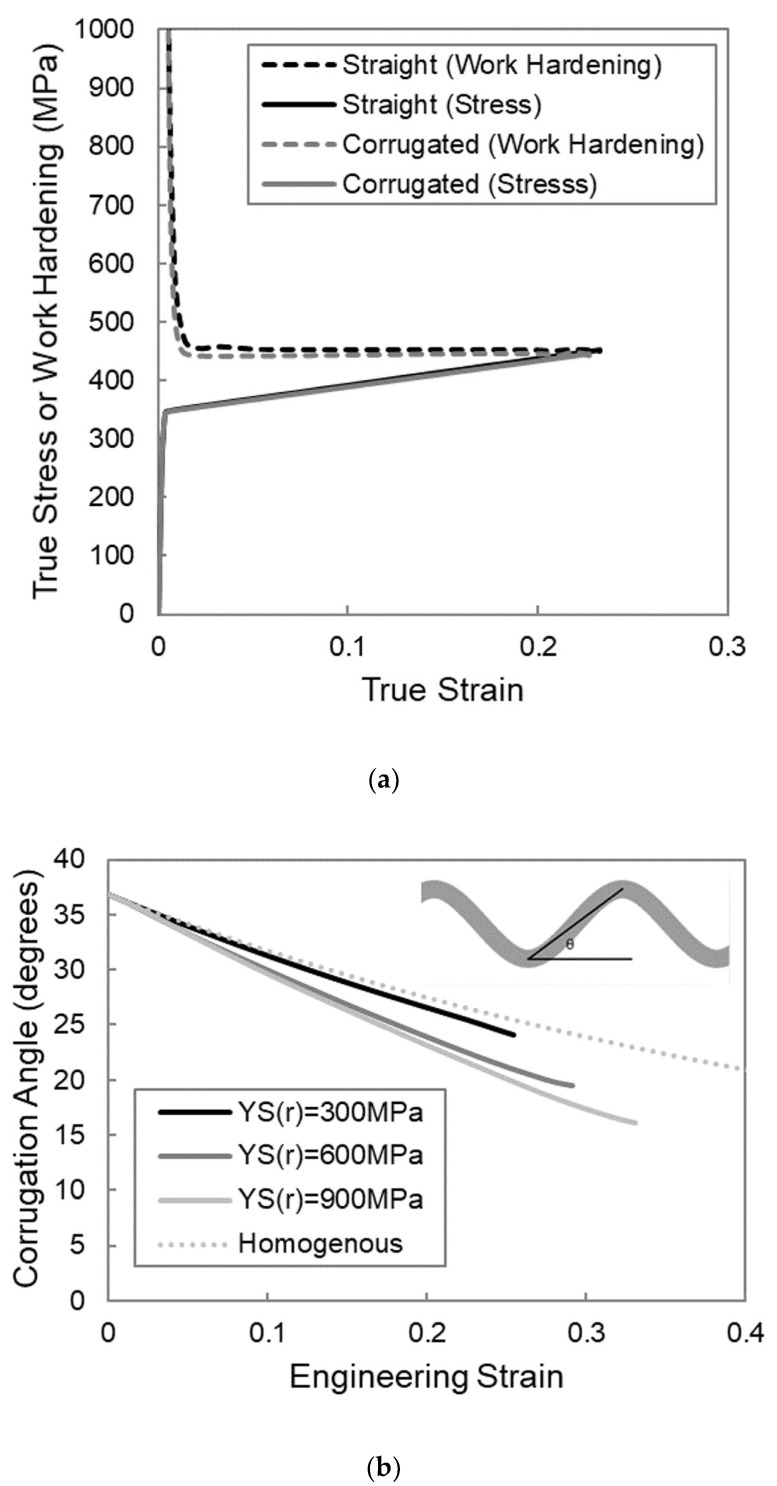
(**a**) Work hardening and stress as a function of strain for both straight and corrugation-reinforced composites that have a low reinforcement yield strength, KS(r), of 300 MPa, which is below the threshold value. (**b**) Evolution of the corrugation angle, θ, for corrugation reinforced composites with different reinforcement yield strengths, KS(r). This plot also includes the angle evolution for a homogenous system in which the corrugation is the same material as the surrounding matrix.

Looking at the work hardening behavior of a corrugated system with a reinforcement yield strength below the threshold value, KS(r) = 300 MPa, as shown in Figure 6a, it is clear that the reason that there is no improvement in necking strain, compared to the straight system, is that there is no boost in work hardening present. This suggests that there is minimal unbending that occurs in these cases. Without unbending, the boost in work hardening will not occur, and thus, there is no impetus for an increase in necking strain. In order to confirm that a lack of unbending is the reason for this lack of benefit, it is instructive to investigate the evolution of the corrugation angle. The corrugation angle can be thought of as a measure of the degree of corrugation and is defined as the angle between the line that connects a peak to an adjacent trough and the horizontal axis, as illustrated in the top right corner of Figure 6b. This figure tracks the corrugation angle as a function of strain for a case in which the reinforcement yield strength is below the threshold, KS(r) = 300 MPa, for a case above the threshold value, KS(r) = 600 MPa, for a case well above the threshold, YS = 900 MPa, and for the limiting case of a homogenous material for which the corrugation is the same material as the matrix and the change of angle is strictly due to the shape change of the sample. The angle evolution for the case with the reinforcement with a yield strength of 300 MPa closely follows the behavior of the homogenous curve, confirming minimal corrugation unbending. This is contrasted with the cases with the higher reinforcement yield strengths, which show a more rapid reduction in corrugation angle, suggesting that the corrugation is unbending to some degree. When the reinforcement yield strength is too low, the region of the reinforcement between the peaks and troughs yields very early on and initiates stretching dominated behavior. Once this stretching is initiated, minimal additional unbending will occur. In order to have unbending occur, the reinforcement yield strength must be high enough that the load that is transferred from the matrix is insufficient to cause reinforcement yielding while the corrugation is undergoing bending dominated behavior at the peaks and troughs.

For the straight reinforced composite, increasing the reinforcement yield strength has a similar effect to increasing the matrix yield strength, i.e., a reduction in the necking strain. For the corrugated composite, on the other hand, once above a threshold value, increasing the reinforcement yield strength leads to increases in the necking strain. This increase in the corrugated composite necking strain is because the higher yield strength delays the point at which the region of the reinforcement between the peaks and troughs yields. This allows the corrugation to unbend to a larger degree, as is evident by comparing the 600 and 900 MPa curves in Figure 6b, resulting in a larger boost in work hardening.

The effect of the final parameter, the reinforcement hardening rate, seems to be much less influential than the others, as shown in Figure 3d. Increasing the reinforcement hardening rate leads to increasing the necking strain for both straight and corrugated composites, with a more drastic increase for the corrugated composites.

To summarize the above findings, in order for a corrugation-reinforced composite to have a larger necking strain than a straight-reinforced composite made from the same component materials, it needs to:

- Have a matrix with a high enough yield strength (or low enough hardening rate) such that the boost in work hardening from the unbending corrugation is not too late.
- Have a matrix with a low enough yield strength (or high enough hardening rate) such that the matrix does not neck prematurely regardless of the geometry and allows unbending to provide a boost in work hardening.
- Have a reinforcement yield strength that is sufficiently high such that the region between the peak and trough does not yield early and begins to stretch, preventing the corrugation from unbending.

As is clear from this summary, the key to utilizing these corrugated composites is ensuring that the corrugation unbends and that this unbending provides a boost in work hardening at the right time. It should be noted that changing the height or period of the corrugation can lead to a boost in work hardening at a different level of strain and thus a combination of materials that is incapable of benefiting from a corrugation with one geometry may be able to boost its necking strain by using a corrugation with a different geometry.

In order to be able to identify whether a composite system is capable of improving its necking strain through the use of a corrugated reinforcement, it would be beneficial to have maps of property space identifying regions of potential benefit. These maps could show the potential improvement in necking strain that can be achieved with different combinations of properties. To create a useful map that is easy to read, it will be necessary to have a plot of only two variables while the rest of the parameters are fixed. The map can be a three-dimensional surface or a two-dimensional colored contour plot in which the third dimension/contours is the difference in necking strain for a corrugated composite and a straight composite.

Based on the results of the parametric studies just presented, it seems that one of the variables should be a matrix parameter. Since the matrix yield strength and hardening rate seem to be interrelated, one of these properties is selected, while the other is fixed. For the purposes of this work, the matrix hardening rate was selected as the variable, while the matrix yield strength was fixed. The other variable that was selected was the reinforcement yield strength, as it seems to be important in determining whether the corrugation will unbend. Therefore, the reinforcement hardening rate will be fixed. Finally, for a given map, the geometry and volume fraction of the corrugation must be fixed. With all this said, sets of maps can be constructed in which one of the fixed parameters can be varied from one map to the next. Therefore, a collection of these maps should provide a good picture of whether a given arbitrary system will benefit from a given corrugated reinforcement geometry.

**Figure 7:**
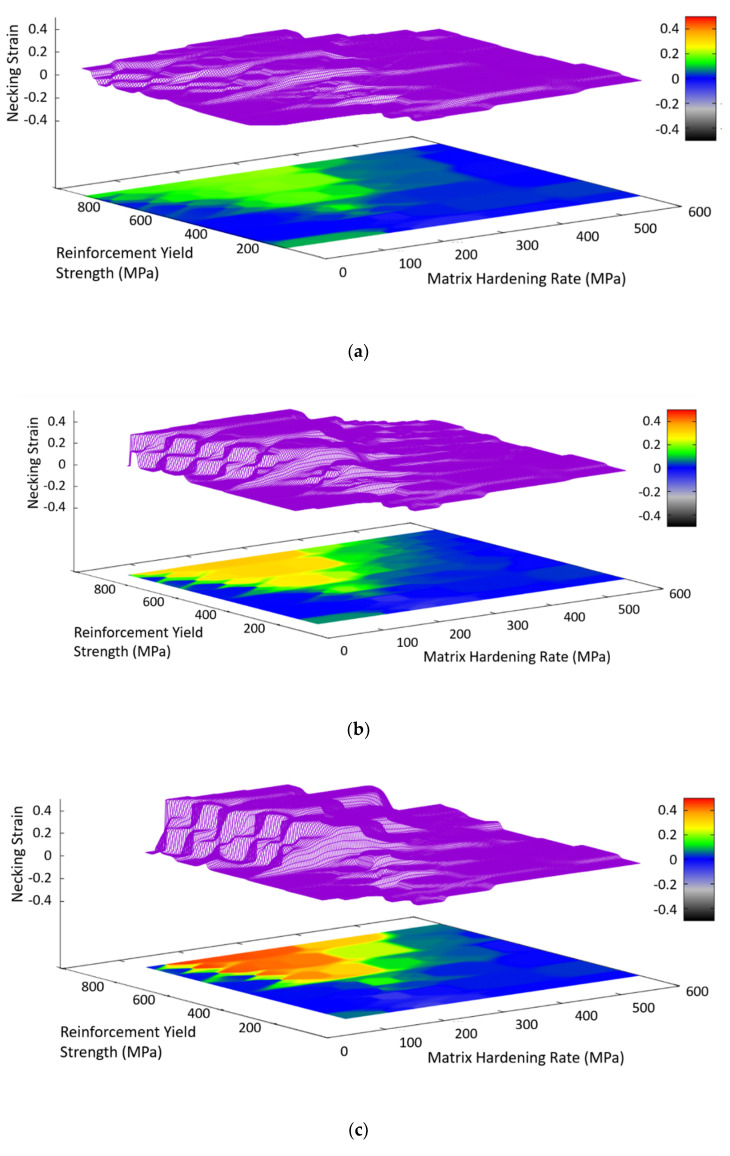
Plots of the difference in necking strain between corrugation reinforced composites and their straight reinforced composite counterparts over a range of values of reinforcement yield strength and matrix hardening rate. Each plot corresponds to a different corrugation height, h; (**a**) 2 mm, (**b**) 3 mm, and (**c**) 4 mm. For all of the simulations represented by these plots, the matrix yield strength was kept constant at 300 MPa, the reinforcement hardening rate was fixed at 500 MPa, and the corrugation geometry had a fixed period of 8 mm and a volume fraction of 20%.

A set of these maps was created for these linear hardening systems by running FEM simulations of straight and corrugated composites across a range of combinations of matrix hardening rate and reinforcement yield strength. The matrix yield strength was fixed at 300 MPa, the reinforcement hardening was fixed at 500 MPa, and the corrugation geometry had a period of 8 mm and a volume fraction of 20%. Three maps were generated, each with a different corrugation height, using the gnuplot graphing software, as shown in Figure 7. These plots contain both a surface, where the height indicates the benefit from corrugation (defined as the increase in necking strain relative to the composite containing a straight reinforcement) and a color contour map, showing the same information. Using a set of maps with different corrugation heights, it is possible to interpolate between the maps in order to predict the behavior for any value of corrugation height. By creating similar sets of maps in which the matrix yield strength or reinforcing hardening rate are varied between the maps in a given set, it should be possible to estimate the benefit through the corrugation of any corrugated composite with linear hardening materials and a 20% volume fraction of reinforcement.

**Figure 8:**
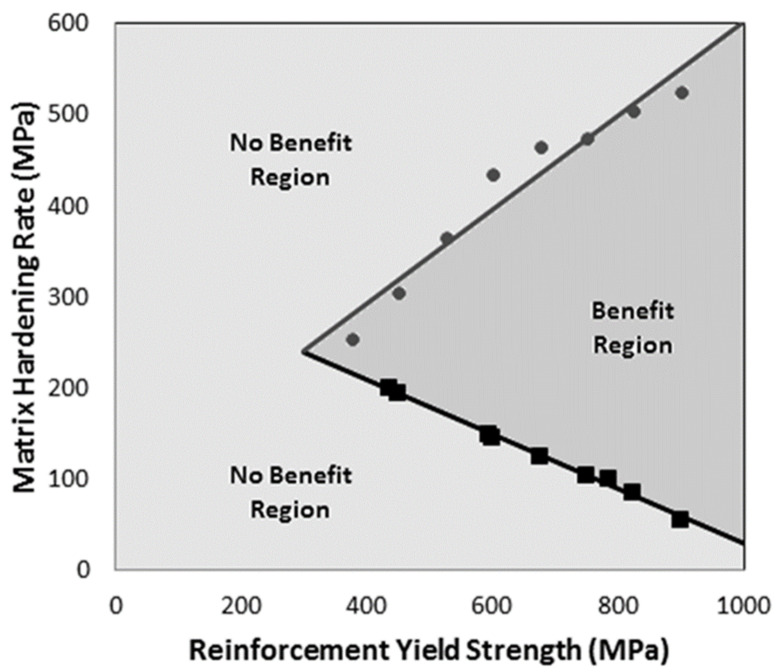
Benefit map showing regions of material property values in which gains in necking strain are achieved through corrugation. This map represents a system with a fixed matrix yield strength at 300 MPa and a reinforcement hardening rate at 500 MPa along with a constant corrugation geometry with a corrugation height of 3 mm, period of 8 mm, thickness of 0.78 mm, and volume fraction of 20%.

An alternative map that could also be useful would divide the property space into two regions; one in which there is benefit through corrugation and one where there is no benefit through corrugation. An arbitrary value for the difference in necking strain needs to be selected that represents the border between the two regions. Using a border value of 0.05 (i.e., a minimum increase in necking strain of 0.05) and plotting for variables of K(m) and KS(r) while fixing the other parameters as YS(m) = 300 MPa, K(r) = 500 MPa and h = 3 mm, one can generate such a property map for 20% volume fraction, as seen in Figure 8. The points on the map represent property combinations that show a benefit through the corrugation of 0.05, and the lines are just fitted to these points. Although this map does not provide information as to the magnitude of the benefit, it is easier to read than the benefit surfaces shown in Figure 7, and it can be used to identify whether a given system will provide a benefit. By creating sets of property surfaces, as shown in Figure 7, and maps, as shown in Figure 8, it would be possible to provide a good estimate as to whether any given system will be able to improve its necking strain by using a corrugated reinforcement geometry.

## 5. Conclusions

The ability of the corrugated reinforcement architecture to improve the necking strain of a composite material is not universally applicable and is dependent on the relative material properties of the matrix and reinforcement materials. Whether a composite system will improve its necking strain through corrugation is dependent first on whether the corrugation is capable of unbending and second on whether the boost in work hardening that comes from that unbending happens at the right time in the deformation process. For the linear hardening systems modeled in this study, it was determined that there is a range of acceptable matrix yield strengths and matrix hardening rates that are capable of improving the necking strain through corrugation. If the matrix yield strength is too low (or the matrix hardening is too high), the boost in work hardening comes in too early, and both the straight and corrugated composites show similar, large values of necking strain. On the other hand, if the matrix yield strength is too high (or the matrix hardening is too low), the matrix material will neck early regardless of the reinforcement geometry, and no unbending of the corrugation will occur. Another finding was that there is a threshold value of reinforcement yield strength that needs to be exceeded in order for a benefit through corrugation to be observed. If the reinforcement yield strength is below this threshold value, the corrugation will stretch instead of unbending, and there will be no boost in work hardening to postpone necking. Finally, benefit surfaces and maps were created with the purpose of outlining the areas of material property space that can benefit through corrugation. By compiling sets of these benefit tools, it should be possible to predict the benefit obtainable if one uses a given corrugation geometry for a given combination of matrix and reinforcement materials.

## Figures and Tables

**Table 1:** Summary of the material parameters used in the FEM studies. Linear work-hardening was assumed for both the matrix and reinforcement. The properties of the matrix and reinforcement are identified by adding (m) and (r), respectively, after the variable name.

Simulation Group	A	B	C	D
Matrix Yield Strength, YS(m) (MPa)	**50**–**400**	300	300	300
Matrix Hardening Rate, K(m) (MPa)	200	**100**–**600**	300	100
Reinforcement Yield Strength, YS(r) (MPa)	600	600	**150**–**1200**	600
Reinforcement Hardening Rate, K(r) (MPa)	500	500	500	**500**–**3000**
